# Investigating the Adoption of Mobile Health Services by Elderly Users: Trust Transfer Model and Survey Study

**DOI:** 10.2196/12269

**Published:** 2019-01-08

**Authors:** Fanbo Meng, Xitong Guo, Zeyu Peng, Kee-Hung Lai, Xinli Zhao

**Affiliations:** 1 eHealth Research Institute School of Management Harbin Institute of Technology Harbin China; 2 Logistics and Maritime Studies Faculty of Business Hong Kong Polytechnic University Hong Kong China (Hong Kong); 3 Department of Information Systems School of Business East China University of Science and Technology Shanghai China

**Keywords:** mobile health, trust, health services for the elderly, adoption, health behavior

## Abstract

**Background:**

Although elderly users comprise a major user group in the field of mobile health (mHealth) services, their adoption rate of such services is relatively low compared with their use of traditional health services. Increasing the adoption rate of mHealth services among elderly users is beneficial to the aging process.

**Objective:**

This study aimed to examine the determinants of mHealth service use intentions using a trust transfer model among elderly users facing declining physiological conditions and lacking support from hospitals.

**Methods:**

A survey comprising 395 users aged 60 years and above was conducted in China to validate our research model and hypotheses.

**Results:**

The results reveal that (1) trust in mHealth services positively influences use intentions, (2) trust in offline health services positively influences trust in mHealth services, (3) declining physiological conditions strengthen the effects of trust in offline health services regarding trust in mHealth services, (4) support from hospitals weakens the effects of trust in mHealth services on use intentions, and (5) the relationship between trust in offline health services and intention to use mHealth services is partially mediated by trust in mHealth services. The independent variables and moderators collectively explain a 48.3% variance in the use intention of mHealth services.

**Conclusions:**

We conclude that the trust transfer theory is useful in explaining the development of initial trust in mHealth services. In addition, declining physiological conditions and support from hospitals are important factors for investigating the adoption of mHealth services among elderly users.

## Introduction

### Background

With advances in health care assisting more people to live longer, the number of people aged over 60 years is projected to reach nearly 2.1 billion, representing 22% of the world’s total population [1]. In China, the proportion of the population aged 60 years and above will increase from 12.4% (168 million) in 2010 to 28% (402 million) in 2040 [2]. This demographic change further intensifies the conflict between the medical care demands of elders and the limited medical care resources. Indeed, mobile health (mHealth) has the potential to enable the elderly to experience longer and healthier lives by transforming health care services and clinical interventions for elderly users [3]. mHealth services are believed to be beneficial for elderly people because they can bring about multiple positive outcomes, such as health care costs savings, individually tailored health information and services, and a more effective health service process [4,5]. Therefore, mHealth is regarded as an important approach of extending the traditional (ie, offline) health services and satisfying the medical demands of an increasingly aging population.

However, older people are often found to be less innovative toward mHealth services [6]. There is limited usage of mHealth services to manage chronic diseases among the elderly [7]. In China, the development of mHealth services continues to remain at the infancy stage, and the adoption rate of such services among older people remains low [8]. The low adoption rate of mHealth services by the elderly has prompted the Chinese government to reconsider their developing strategies, to encompass the rapidly growing number of elderly users to alleviate the pressures of the aging population. In addition, although the elderly could obtain great potential benefits by using health information technology (HIT), previous research has not examined the relationship between HIT use among elderly users from their use of health services [9,10].

Due to their unique physical and psychological characteristics, elderly users may need to expend much more effort and time familiarizing themselves with information technology (IT) than the younger user groups [11]. In addition, declining physiological conditions [12] and limited social resources [13] are hurdles for elderly individuals in making health-related decisions independently. To serve the elderly population through mHealth services, service providers need to understand the behavior of this special group and the antecedents that influence their acceptance and usage of mHealth.

Building user trust is the key to promoting the adoption of mHealth services by elderly users. Trust could alleviate uncertainties and risks when users encounter new IT or services (eg, purchasing books on the Web and mobile payment services) [14,15]. In this context, trust transfer is a means of building customer trust in an unknown target through a trusted party [16]. Customer trust can be developed through trust transference from offline health care services, which are trusted by the elderly, to mHealth services with which they are not familiar. Although trust plays an important role in mHealth adoption, very few previous studies have investigated the development of trust in mHealth services [17,18]. Furthermore, as mHealth services are developed based on the traditional service in a new context, that is, a mobile context, users’ perceptions on mHealth may be influenced by the offline traditional services, such as trust. However, research on the trust transfer from an offline context to a mobile channel is also underexplored in both the information system (IS) and health care fields. In addition, although considerable research on trust transfer has been conducted in the IS domain, inadequate research has been undertaken on trust transfer in mHealth research.

Our study aims to develop a trust transfer model to investigate the adoption of mHealth services by the elderly and to address the following research question: To what extent is trust transfer a means of establishing the initial trust of elderly users in their adoption of mHealth services? In this study, based on the trust transfer theory, we incorporate both declining physiological conditions and support from hospitals into our research model to explore their moderating effects on the trust transfer process. To validate the research model and proposed hypotheses, a survey comprising 395 elderly users in China was conducted to analyze the research model.

### Literature Review and Hypotheses

#### Trust Transfer

Trust can develop through a transference process, suggesting that trust can be transferred from a trusted object to an unfamiliar object [19]. Channels of trust transfer include the intrachannel and interchannel transfers [20]. An intrachannel trust transfer is one where trust can be transferred within the same channel that is deemed as trustworthy. For example, trust transfer can occur either from offline to offline contexts or from Web to Web contexts. Stewart illustrated that trust can be transferred from a trusted hypertext link to an unfamiliar one on a website [16]. In fact, Stewart also suggested that trust transfer may work based on a cognitive process that is based on the mere knowledge of the relationship between the trusted target and trusted source.

On the other hand, interchannel trust transfer refers to trust transfers from one context to another, mainly from the offline to the Web channels or from the Web to mobile channels. Turel et al indicated that in a transfer from an offline to a Web channel, the e-customer service provider can improve user trust and use intention by associating itself with a known human service representative [21]. Belanche et al purported that trust in offline public administration recommendations can be transferred to trust in the online public e-service [22]. From the perspective of transfer from a Web to a mobile channel, the level of trust in internet payment services is positively associated with the initial trust in mobile payment services offered by the same company [23]. Similarly, in a study on brokerage services, trust in an online environment is positively related to initial trust in a mobile environment [20]. On their part, Wang et al studied the mobile e-word of mouth (eWOM) services and found that trust in Web services has a positive effect on trust in mobile services, thus influencing the intention to use mobile eWOM services [24].

Offline health services are the pivotal source of health services for the elderly. These users’ prior experiences and familiarity with offline health services have resulted in the development of their deeply ingrained belief that offline health services are trustworthy. Accordingly, in the interchannel trust transfer context, we anticipate that trust in well-established offline health services would affect trust in corresponding mHealth services.

Thus, we hypothesize the following:

Hypothesis 1: Trust in offline health services is positively associated with trust in mHealth services.

#### Trust in Mobile Health Services

Prior research demonstrates that the conceptualizations of trust are diverse, incomplete, and inconsistent [25]. Integrating with the shared characteristics of trust across different disciplines, Mayer defined trust as “the willingness of a party to be vulnerable to the actions of another party based on the expectation that the other will perform a particular action important to the trustor, irrespective of the ability to monitor or control that other party” [26]. According to prior research, beliefs in competence, integrity, and benevolence lead to a general belief in trust and behavioral intentions [14,27,28]. All these 3 main trusting beliefs are the trustee’s internal trust-related characteristics observed by the trustor, and these trusting beliefs are formatted in the cognitive process of a trustee’s attributes.

As trust can reduce risks and uncertainties, trust plays a significant role in the adoption of a new IT [29]. Prior studies underscore that trust is a significant prerequisite of social behavior and positively associated with users’ use intentions of an internet store [27], purchasing books on the internet [14], e-government services [30,31], e-commerce [25,32,33], and mobile payment [34].

From the perspective of mHealth services, Guo et al found that trust in mHealth service providers enables the reduction of individuals’ privacy concerns and an increase in adoption intentions [18]. Zhao et al indicated that trust is positively associated with the behavioral intention to use mHealth services [35]. Deng et al demonstrated that patients’ trust positively affects the adoption intention of mHealth services [36]. On the basis of their findings of the post adoption stage, Akter et al theoretically conceptualized trustworthiness (trusting belief) in mHealth service research and indicated that trustworthiness positively influences consumer trust (trusting intentions), which directly affects consumers’ continuance intentions [17]. In a later research, Akter et al demonstrated that perceived trust positively affects satisfaction with mHealth services and continuance intention [37].

As the mHealth service is a credence product and personalized service, trust plays a significant role in predicting individuals’ adoption intentions. Elderly users, in particular, have less experience with the use of mHealth services and encounter more difficulties in using this emerging technology. As health is a sensitive subject, these users may pay more attention to health services assessed through mobile channels.

Thus, we hypothesize the following:

Hypothesis 2: Trust in mHealth services is positively associated with the intention to use mHealth services.

#### Declining Physiological Conditions

Individuals’ physiological conditions (such as hearing, vision, speech, locomotion, and memory capabilities) are known to decline in the aging process, thus influencing their physical and cognitive capabilities [38]. Timmermann demonstrated that the declining physical and cognitive capabilities may cause elders to experience considerably greater difficulty in the use of computers, and these declining physiological conditions can serve as internal controls or inhibiting conditions that increase the effort expectancy associated with IT use [39]. Mathur indicated that age-related decline in physiological conditions will increase the elderly’s need for family assistance in coping with these declines and revealed that family assistance had a positive effect on the adoption of IT [13].

Phang et al introduced new constructs that are used in gerontology and IS literature, comprising preference for human contact, self-actualization, and resource savings as antecedents of perceived usefulness, whereas computer anxiety, computing support, and declining physiological conditions reflect perceived ease of use in the research of e-government service use intentions [12]. Heart and Kalderon demonstrated that cognitive and physical impairments that increase with age are negatively associated with health-related IT use [40].

Xue et al integrated aging-specific constructs including perceived use resources, technology anxiety, and bio-physical age (perceived physical conditions) with the technology acceptance model to reveal that perceived use resources and technology anxiety were antecedents for perceived usefulness, whereas perceived physical conditions significantly influenced perceived ease of use [41]. Deng et al found that elderly users’ aging characteristics, including declining physiological conditions, technology anxiety, and self-actualization needs, positively influence their use intentions of an mHealth service [8].

Therefore, declining physiological conditions may cause elderly users to expend greater energy on evaluating the competence, reliability, and dependability of mHealth services. Hence, elderly users are more likely to develop trust in mHealth services based on trust in offline health services.

Thus, we hypothesize the following:

Hypothesis 3: The relationship between trust in offline health services and trust in mHealth services will be stronger in the case of declining physiological conditions.

#### Support From Hospitals

On the basis of the previous literature on IT acceptance and usage, the source of support is mainly an organization [42] or an expert user [43]. An organization can offer managerial interventions and support resources to encourage users to accept IS and improve performance outcomes [44]. In their study of personal computer usage, Compeau and Higgins observed that an organization’s support for computer users influences individuals’ judgments of self-efficacy because support and assistance from an organization assisted users in increasing their ability [45]. Hoque and Sorwar found that elderly users believe an organizational support or technical infrastructure support positively affect their behavioral intentions toward mHealth services [46]. In the context of the adoption of mHealth services, with support from hospitals, elderly users are able to efficiently evaluate the competence of mHealth services and develop trust in mHealth services. This leads to an intention to use mHealth services. When elderly users receive support from hospitals, they are more likely to develop a high level of trust in mHealth services.

**Figure 1:**
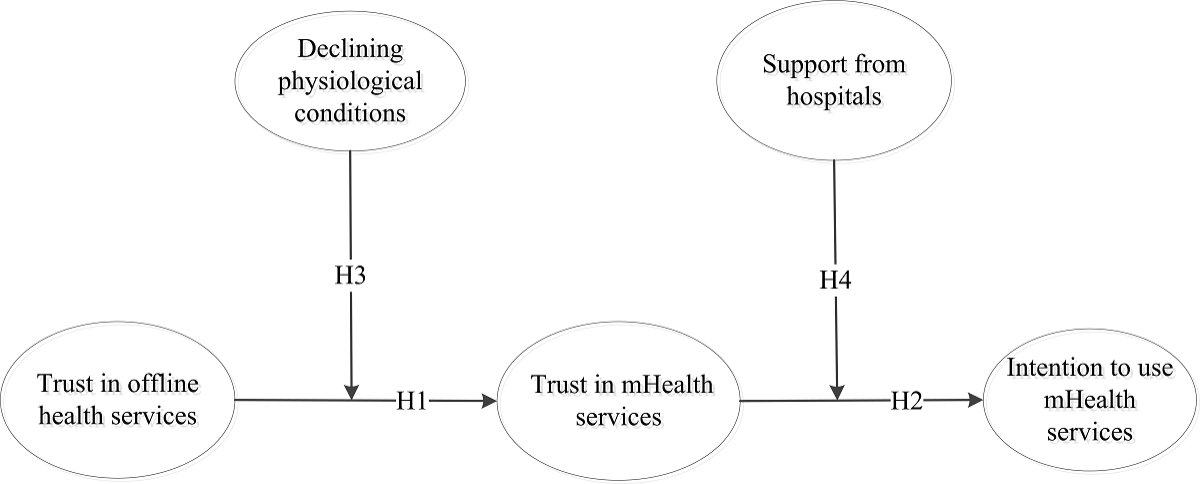
Research model. mHealth: mobile health.

Thus, we hypothesize the following:

Hypothesis 4: The relationship between trust in mHealth services and the intention to use mHealth services will be stronger when hospital support is provided.

In summary, this study proposed a trust transfer model to predict elderly users’ intentions to use mHealth services, as depicted in Figure 1. According to the trust transfer theory [16,47], trust in offline health care services positively influences trust in mHealth services. A declining physiological condition, as a salient variable of gerontology, influences the trust transfer process from an offline channel to a mobile channel. In addition, support from hospitals has a moderating effect on the relationship between trust in mHealth services and use intentions.

## Methods

### Development of the Study Questionnaire

All the measures in the survey were adapted or adopted from existing prevalidated instruments. The measures of trust in offline health services and trust in mHealth services were adapted from the study by Gefen et al [25]. The intention to use the mHealth service was measured using the items from the study by Deng et al [8]. The measures of declining physiological conditions were adapted from the study by Phang et al [12]. The measures of support from hospitals were adapted from the measures of vendor support in the study by Thong et al [48]. In fact, Thong et al indicated that vendor support results in a higher level of IS effectiveness. In the context of our study, the hospital of our study launched an mHealth service to extend the traditional offline health services. The role of the hospital is very similar to the role of the vendor. Therefore, we modified the measurement items of vendor support according to our research context.

To improve the content validity of the questionnaire, we revised the questions and deleted similarly phrased items based on feedback from 2 mHealth researchers and a pretest with 30 elderly users. All items included in the survey were measured on a 7-point Likert scale ranging from 1 to 7, with “1” representing strongly disagree and “7” representing strongly agree. The details of the constructs and measurements are presented in Multimedia Appendix 1.

### Data Collection

We conducted our survey at Aerospace Center hospital in Beijing, the capital of China. At the end of 2016, this hospital had launched a health management platform aiming to provide reliable daily health care services for patients through mobile services. Therefore, this target hospital is an appropriate site for data collection. The mHealth services provided by this platform included a routine appointment out-patient clinic, follow-up visits, medicine reminders, medical records, and real-time positioning. Permission was obtained, and proper arrangements were made by the management board of the hospital for successful data collection. We followed 3 screening criteria to select the target participants: (1) patients coming to a physical examination center for medical examination, from which we could assume that they cared about their health; (2) patients who did not have a serious disease; and (3) patients aged 60 years or above. As these elderly participants could find it difficult to complete questionnaires unaided because of literacy problems, we accordingly employed 6 postgraduate students to assist the participants in completing the questionnaires. Furthermore, considering that most of the participants might be unfamiliar with mHealth services, a copy of an instruction manual of mHealth services was shown to the participants before they completed the questionnaire.

In fact, 500 copies of the survey questionnaire were distributed, and 395 usable copies were obtained, accounting for a response rate of 79.0% (395/500). We excluded 105 participants because, owing to several reasons, they were not our target subjects. First, these participants were illiterate. Second, their declining abilities regarding reading, writing, and speaking hindered their use of mobile services. Third, these participants did not own mobile phones, which could support the use of mHealth services. Accordingly, we selected the remaining respondents as our target subjects to use in the data analysis. Among these participants, approximately 49.1% (194/395) were males and 50.9% (201/395) were females. In fact, 316 out of 395 participants (80.0%) were aged from 60 years to 70 years. In addition, 75.9% (300/395) of the participants had attended high school. All respondents were given ¥10 (US $1.5) supermarket coupons for their participation.

### Data Analysis

Partial least squares (PLS) was used to test the research model because of the several advantages of this technique. First, PLS can predict all loadings and weights of indicators and causal relationships among constructs in multistage models [49,50]. Second, compared with covariance-based (CB) structural equation modeling (SEM), PLS is the most suitable technique for models with formative constructs and is appropriate for relatively small samples [51], which is the case in our study. In addition, PLS provides a good approximation of CB-SEM in terms of final estimates [50,51]. Given these considerations, we adopted the PLS to analyze our research model.

The data analysis was conducted in 2 stages. In the first stage, the measurement model (ie, based on the reliability, validity, common method bias, and multicollinearity of the constructs) was assessed to ensure its appropriateness. In the second stage, the structural model was examined and the stated hypotheses were tested [52].

## Results

### Results of the Measurement Model Testing

The internal reliability, convergent validity, and discriminant validity were examined to assess the measurement model [53]. The reliability of the constructs was tested using Cronbach alpha, composite reliability (CR), and average variance extracted (AVE) [54]. In our study, the threshold values of the CR and AVE were .70 and .50, respectively, which is consistent with the work by Chin [55]. The value of Cronbach alpha, which was greater than the threshold .70, indicated adequate construct reliability [56]. CRs for these constructs ranged from .906 to .957, and the AVE varied from .765 to .881. All the values of Cronbach alpha and CR were above the threshold values, indicating good construct reliability [54]. All the item loadings of each construct were significant and above the suggested cut-off value (.700), indicating convergent validity [55]. As shown in Table 1, the loadings of all items were much greater than the cross-loadings on other constructs, and the correlations of any 2 constructs were significantly smaller than the square root of the AVE of each construct, further indicating acceptable discriminant validity. In addition, we tested the potential issue of multicollinearity through the use of the variance inflation factor (VIF). As a rule of thumb, the threshold value of the VIF is less than or equal to 10, indicating no presence of multicollinearity. The results indicate that all VIFs are less than 7, thereby suggesting that there is no multicollinearity among or between the independent variables.

Common method bias could be a potential concern as the data were collected from a self-reported survey [57]. We first tested for common method bias according to Harman single-factor test [58]. According to the results, we found that the factors accounted for 81.5% of the variance and the first factor only explained 25.1% of the variance, thus indicating that common method bias was not likely to have been an issue.

### Results of the Structural Model Testing

**Figure 2:**
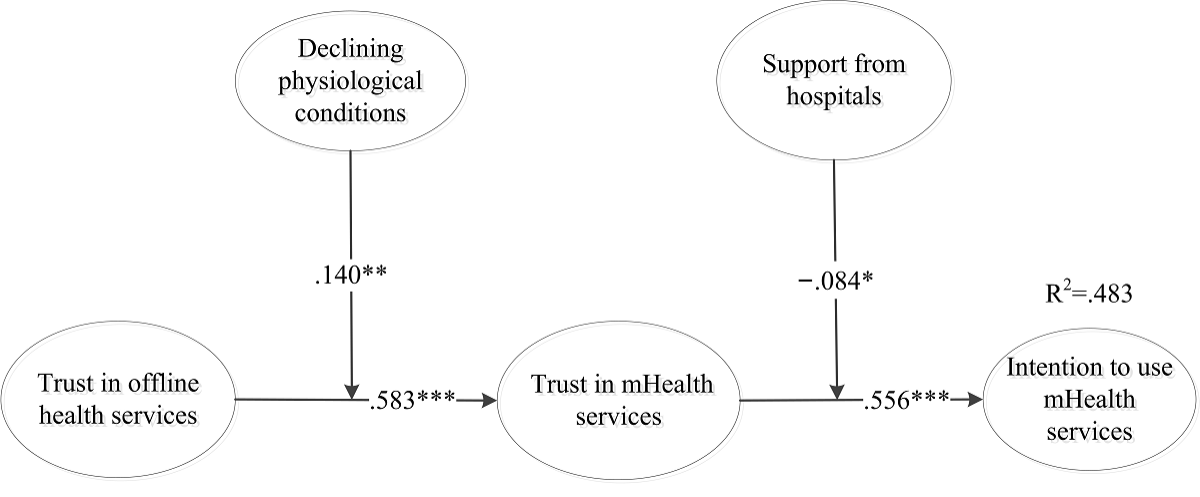
Results of the research model. mHealth: mobile health. Asterisk indicates *P*<.10; two asterisks *P*<.01; three asterisks *P*<.001.

The results of the structural model are recorded in Figure 2. They indicate that trust in mHealth services (beta=.556; *t*_394_=11.174; *P*<.001) had significant effects on the intention to use mHealth services. Hence, hypothesis 1 is supported. The results demonstrate that trust in offline health services has a significant effect on trust in mHealth services (beta=.583; *t*_394_=14.528; *P*<.001), thus supporting hypothesis 2.

The moderating effects of declining physiological conditions and support from physicians were further tested. Declining physiological conditions were perceived to have a positive moderating effect on the relationship between trust in offline health services and trust in mHealth services (beta=.140; *t*_394_=2.723; *P*=.003), thus lending support to hypothesis 3.

**Table 1:** Correlations and discriminant validity.

Construct	Cronbach alpha	Composite reliability	AVE^a^	Use intention	Trust in offline health services	Trust in mHealth^b^ services	Declining physiological conditions	Support from hospitals
Use intention	.888	.930	.816	.903^c^	—^d^	—	—	—
Trust in offline health services	.932	.957	.881	.531	.938^c^	—	—	—
Trust in mHealth services	.914	.945	.853	.683	.582	.923^c^	—	—
Declining physiological conditions	.915	.906	.765	.033	−.007	.052	.874^c^	—
Support from hospitals	.929	.949	.824	.535	.581	.682	.093	.907^c^

^a^AVE: average variance extracted.

^b^mHealth: mobile health.

^c^Square root of average variance extracted.

^d^Not applicable.

**Table 2:** Summary of the hypotheses testing.

Hypothesis	Description	Path coefficient	*P* value	Supported
1	Trust in offline health services is positively associated with trust in mHealth^a^ services.	.583	<.001	Yes
2	Trust in mHealth services is positively associated with intention to use mHealth services.	.510	<.001	Yes
3	The relationship between trust in offline health services and trust in mHealth services will be stronger in the case of declining physiological conditions.	.140	.003	Yes
4	The relationship between trust in mHealth services and intention to use mHealth services will be stronger in the case of support from hospitals.	−.084	.04	No

^a^mHealth: mobile health.

Support from hospitals was seen to negatively moderate the relationship between trust in mHealth services and intention to use mHealth services (beta=−.084; *t*_394_=1.725; *P*=.04). Hence, hypothesis 4 is not supported.

In addition, the mediating effect of trust in mHealth services in the proposed model was tested according to the procedures provided by Baron and Kenny [59]. First, the relationship between trust in offline health services and intention to use mHealth services was tested (beta=.532; *t*_394_=10.252; *P*<.001). Then, we tested the relationship between trust in mHealth services and trust in offline health services (beta=.582; *t*_394_=13.191; *P*<.001). Finally, We tested the relationship between trust in offline health services and intention to use mHealth services (beta=.202; *t*_394_=3.420; *P*<.001), and the relationship between trust in mHealth services and intention to use mHealth services (beta=.566; *t*_394_=11.988; *P*<.001). Therefore, the relationship between trust in offline health services and intention to use mHealth services is partially mediated by trust in mHealth services. The results of each hypothesis are summarized in Table 2.

## Discussion

### Principal Findings

This study yields several important findings. First, trust in mHealth services positively affects elderly users’ use intentions. This highlights the proposition that trust in mHealth services is of importance in predicting the adoption of mHealth services. Second, trust in offline health services has significant effects on trust in mHealth services, which indicates that trust in offline health services can be transferred to the mobile environment. This suggests that mHealth service providers may be able to swiftly build elderly users’ trust in mHealth services through leveraging their existing trust in offline health services, thus leading to a higher adoption rate of mHealth services. Third, declining physiological conditions are seen to strengthen the relationship between trust in offline health services and trust in mHealth services. As physiological capabilities decline with age, elderly users are less capable of using new IT apps. As elderly users with declining physiological conditions need to exert more effort to evaluate the competence and ability of mHealth services, they rather tend to rely more on their prior experience and knowledge of offline health services to cultivate trust in mHealth services. Fourth, support from hospitals is seen to weaken the association between trust in mHealth services and the intention to use mHealth services. Contrary to our hypotheses, support from hospitals decreases patients’ trust in mHealth services because they depend more on physicians’ support instead of expending extra effort on evaluating the competence and ability of mHealth services. There is possibly a mismatch between their perceptions and their physicians’ advice.

### Theoretical and Practical Implications

This study can enrich and advance our theoretical understanding in several ways. First, it extended the trust transfer theory to the offline context in transition to mHealth services. Previous studies of the trust transfer theory have mainly focused on the perspectives of offline to online and online to mobile channels [16,20,22,60]. On our part, we explored the trust transfer process in the context of the new mHealth services from a cross-environment perspective. As the offline to mobile service transition has become a trend for most offline services, our research into this phenomenon provides a valuable reference. Our study can be seen as an attempt to fill this research gap by providing a cornerstone for further theoretical development.

Second, to comprehensively understand the IT acceptance behavior of the elderly, this research introduced a variable from gerontology—declining physiological conditions. Previous IS research on the IT acceptance behavior of a specific group is rare, except for the studies by Phang et al [12] and Deng et al [8]. On our part, the moderating effect of declining physiological conditions was tested in the trust transfer from offline to mobile channels. Indeed, exploring the moderating role of declining physiological conditions on trust transfer will facilitate the understanding of which conditions are more effective and thus further extend our knowledge of the trust transfer theory.

Third, support from hospitals was introduced in the context of research on mHealth services. Social support is positively associated with health behavior in the health care literature [61-63]. However, studies on the role of social support in moderating the associations between these 2 trust elements and intention to use have been limited. We suggest that future studies should take social support into account in an attempt to better understand how trust elements influence elderly users’ attitude changes and health behaviors.

This study also reflects several practical implications. First, the important role of the relationship between offline health services and mHealth services suggests that offline trust can be used as an enabler that encourages a provider of mHealth services to expand from the offline to mobile channels. Accordingly, mHealth service providers are encouraged to increase their cooperation with hospitals that are trusted by patients when marketing their mHealth services to elderly users. This may lead to higher adoption rates than simply promoting mHealth services in isolation.

Second, hospitals should undertake responsibility for ensuring that the elderly use mHealth services because they possess limited health literacy and limited experience with mHealth services. However, hospitals need to provide support to elderly users in a proper way to avoid them being overly dependent on hospitals. This is because elderly users who rely too much on support from hospitals are less likely to develop trust in mHealth services, which can weaken their intention to use mHealth services.

Third, mHealth service providers need to understand the different behaviors among different groups of users of mHealth services. Service providers are urged to employ tailored strategies to promote their mHealth services to elderly users when they develop marketing campaigns targeting the building of trust in a mobile environment.

### Limitations

As with all empirical research, this study has its limitations. First, the study did not include users of all age groups. Elderly users were taken as the sample in our study because this specific group accounts for a large portion of all users of mHealth services. Our results need to be interpreted with caution for applications in other population and age groups. Second, this study was conducted in China, and the results may be applicable only in cultural contexts similar to those of the Chinese mainland. We suggest a similar study in a Western context for comparing the results across different cultures. Third, although the explanatory power of the model is acceptable (48.3% for intention to use), we still advocate the potential to enhance our explanatory power through taking additional factors into consideration, in future research.

### Conclusions

In conclusion, we reiterate that mHealth services are regarded as an essential means to alleviate the conflicts between the medical demands of an increasingly aging population and limited medical care resources. However, the adoption rate of mHealth services among elderly users still remains low. Our research draws upon the trust transfer theory and builds a comprehensive framework by integrating declining physiological conditions and support from hospitals to investigate the initial trust-building mechanism of mHealth services. The results indicate that trust in offline health services has a significant effect on trust in mHealth services, thereby leading to an intention to use mHealth services. Declining physiological conditions is seen to positively moderate the association between trust in offline health services and trust in mHealth services; however, contrary to our hypotheses, support from hospitals weakens the association between trust in mHealth services and the intention to use mHealth services. The results provide a good explanatory power to predict the mHealth use intentions of elderly users. These findings have advanced the trust transfer theory and enriched the literature on mHealth services. Accordingly, mHealth services practitioners can better understand how to leverage the benefits of trust transfer and the characteristics of elderly users to promote their mHealth services.

## Appendix

Multimedia Appendix 1 Questionnaire: measurement of the major constructs.

## Abbreviations

AVE: average variance extracted
CB: covariance-based
CR: composite reliability
eWOM: e-word of mouth
HIT: health information technology
IS: information system
IT: information technology
mHealth: mobile health
PLS: partial least squares
SEM: structural equation modeling
VIF: variance inflation factor
