# Supplementary material for: Investigating the Adoption of Mobile Health Services by Elderly Users: Trust Transfer Model and Survey Study
Source: JMIR Mhealth Uhealth. 2019 Jan 8;7(1):e12269. doi: 10.2196/12269 (PMC6329414; doi:10.2196/12269)
Supplement: Multimedia Appendix 1 [file mhealth_v7i1e12269_app1.pdf]

Multimedia Appendix

Questionnaire: Measurement of the major constructs

Use intention

UI1: I have a strong intention to use mHealth services.

UI2: I intend to learn about using mHealth services.

UI3: I plan to use mHealth services to manage my health.

Trust in the offline health services

TOHS1: I know the offline health service is honest.

TOHS2:I know the offline health services cares about its customers.

TOHS3: I know the offline health service is not opportunistic.

TOHS4: I know that the offline health service is predictable.

Trust in the mHealth services

TMS1: I know that the mHealth service is honest.

TMS2: I know that the mHealth service cares about its customers.

TMS3: I know that the mHealth service is not opportunistic.

TMS4: I know that the mHealth service is predictable.

Support from hospitals

SFH1: Hospitals provide adequate technical support during mHealth implementation.

SFH2: Hospitals provide a high quality of technical support during mHealth implementation.

SFH3: Hospitals provide adequate training during mHealth implementation.

SFH4: Hospitals provide a high quality of training during mHealth implementation.

Declining physiological conditions

DPC1: My physiological conditions require me to exert more effort to perform daily activities.

DPC2: My physiological conditions limit the kind of activities that I can perform.

DPC3: My physiological conditions cause me to have difficulty in performing daily activities.
